# Validation of mouse welfare indicators: a Delphi consultation survey

**DOI:** 10.1038/s41598-019-45810-y

**Published:** 2019-07-15

**Authors:** Ivone Campos-Luna, Amy Miller, Andrew Beard, Matthew Leach

**Affiliations:** School of Natural and Environmental Sciences, Agriculture Building, Newcastle University, Newcastle upon Tyne, NE1 7RU UK

**Keywords:** Animal behaviour, Experimental models of disease

## Abstract

This study aims to identify the most valid, reliable and practicable indicators of laboratory mouse welfare using the Delphi consultation technique. The effective assessment of laboratory mouse welfare is a fundamental legal and moral requirement as it is critical part of both maintaining and improving the welfare of the most widely used laboratory animal globally. Although many different welfare indicators are routinely used to assess mouse welfare, the validity, reliability and practicability of many of these measures remains unclear. The Delphi consultation technique is designed to gauge expert opinion through multiple rounds of surveys until a consensus is reached. Participants ranked 59 welfare indicators in terms their validity, reliability and practicability for either a half-day unit audit or a daily welfare assessment and for each scenario identified 10 key indicators. The Delphi consultation reached consensus at 72% for the overall list of indicators and over 60% for each individual indicator. From this consensus the key indicators for each mouse welfare scenario (half day audit and daily welfare assessment) were identified and used to create a welfare scoring system for each scenario.

## Introduction

Mice are the most commonly used species in scientific research, with over 4.6 million mice estimated to be used annually in regulated research globally^1^. Over 3 million scientific procedures involving animals were carried out in the UK during 2017, 58% of which involved mice^2^. Due to the large number of mice used in research, refinement of their welfare is critical. This refinement is dependent on our ability to efficiently assess their welfare, as without this assessment we cannot identify instances when a refinement is needed or if any refinement applied has been effective. Consequently, there has been increasing interest in developing new methods to effectively assess the welfare of mice at both an individual and group level within animal facilities^3–6^. The welfare of laboratory mice is routinely assessed using a combination of animal-based indicators (e.g. physiological, psychological changes) or resource-based indicators (e.g. environmental conditions, staff training), along with indices derived from the specific procedures or studies (e.g. pain management)^7–10^. Resource-based assessment is carried out using indicators that reflect the animals environment and how animals cope with the environmental changes, preserving their biological and psychological functions. Indicators include environmental indices relating to the animals’ housing and husbandry as well as every day husbandry activities (e.g. cleaning cages)^11^. Animal-based assessment involves the measurement of an animal’s behaviour and physiological reactions. The aggregation of all aspects of laboratory mouse welfare (physical, physiological, behavioural and environmental) into a welfare protocol, is paramount to provide an overall assessment. There have been limited studies gathering information from experts about indicators and methods for assessing animal welfare. These studies were conducted a few years ago (around 2010), and the majority focus on species other than mice, such as cows^12^, horses^13^, pigs^14^ and laying hens^15^ and their focus was the development of policies and recommendations for welfare^16–18^. Most of these studies use the Delphi method which is a widely used survey technique that seeks information from experts about a specific topic^13,19,20^. The answers are given anonymously, through a series of rounds with the aim of achieving consensus within the group^21^. The Delphi methodology has been used in diverse range of animal science fields, including to assess the impact of DEFRA policy on welfare^16^; the implication of animal diseases on productivity^22^; and for the selection of a subset of species to have their habitat protected^23^. This technique has shown to be an effective method of gaining information about welfare assessment in farm animals^13–15^. In these studies, the Delphi consultation process was used in different ways, including the use of vignettes with horse welfare case scenarios^13^, questions about preferences in animal-based welfare indicators for hens, pigs and cattle^15^ and with  case studies in livestock including dairy and egg production^16^. The results of these studies provided information about stakeholders attitudes towards methods of improving horse welfare^13^; formed a foundation for the development of welfare protocols including indicators related to health status, behaviour and records which were selected by the experts (e.g. lameness in dairy cattle)^15^ and highlighted the need to increase of monitoring compliance regarding welfare standards in dairy and egg production systems^16^. The Delphi methodology is used as one of the preliminary sources for assessing ‘face’ validity, which is defined as the subjective opinion of experts about the extent to which the measure is meaningful in terms of providing information on the animal’s welfare^24–26^. This face validity is based on the assumption of “safety in numbers” where a group of people are less probable to come to a wrong conclusion than an individual^27^. This study uses expert opinion about the validity of laboratory mouse welfare indicators.

The aim of this study was to determine, through a modified Delphi consultation, which indicators of mouse welfare are considered valid, reliable and practical for a half-day audit and daily welfare assessment of laboratory mice. This study uses the Delphi consultation technique as a tool to identify potential measures for assessing mouse welfare. In this consultation, a level of 70% global consensus (i.e. across all the indicators) and over 60% individual indicator consensus was required. There are no specific guidelines offering a definition of consensus in Delphi studies, as it is argued it depends on the nature of the research that is carried out (e.g. medical decision, development of new policies etc.)^28^. Studies using this technique in nursing and animal welfare contexts have used a level of 70% consensus as a standard^29–32^. However, many studies do not provide any information about the level of consensus needed^13,16,23^ or if they are required to have 100% agreement^22^. Since there are no guidelines to set a consensus level in Delphi studies a level of 70% was used as this is aligned with other peer-reviewed, animal welfare research^18,33^.

## Results

### Demographics

Of the 98 participants who completed both rounds of the Delphi consultation, 30% were veterinarians, 20% were researchers who used animals in their research, 19% were laboratory facility managers, 11% were technicians, 10% were Named Animal Care Welfare Officers, and 8% were Animal Welfare researchers. The majority of the respondents were working in the United Kingdom (41%), followed by USA (13%), Australia (12%), Canada (10%) and Switzerland (8%). Participant expertise was based on the number of years of experience working with laboratory animals, qualifications and job position. Most of the participants had a PhD (35%), other qualifications related to animal welfare (e.g. IAT, Diploma in animal science) (20%) or a Masters in Animal Behaviour and Welfare degree (15%).

### Half day welfare audit

**Figure 1:**
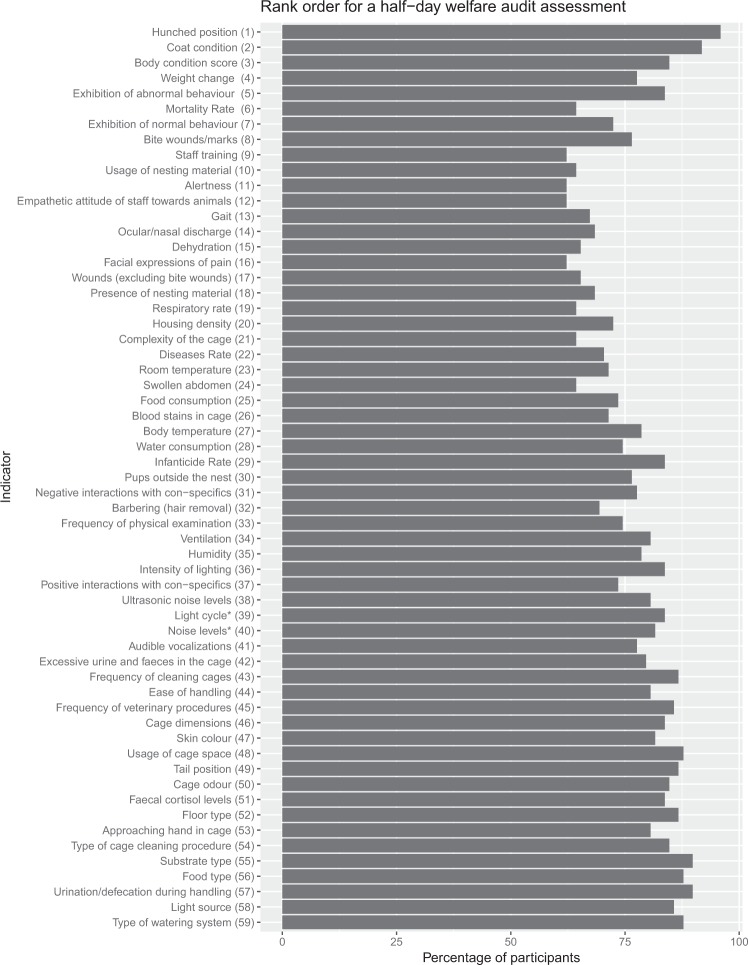
The mean rank order for mouse welfare indicators after round two of the consultation process. Percentage of participants who chose the assigned rank order +/−2 positions are indicated to the right of the figure.

A total of 98 participants completed the second-round questionnaire. Twenty-nine percent of these participants agreed to the initial rank order (from round one) and seventy-one percent of participants made minor changes to the indicators rank order from round one (Fig. 1). The rank position of the indicators was not modified significantly as the order of the indicators did not change from one extreme position to another, although a lower level of agreement can be seen in some of the indicators (e.g. alertness and staff training, both with 62% agreement). The overall consensus for the rank order of indicators used in a half-day audit assessment was 77.2%. Based on these results, a consensus among the participants was reached in the second round so no further consultation was deemed to be necessary. The indicators with the highest level of consensus were hunched position and coat condition ranked first and second with over 90% of agreement between participants. The indicators with the lowest consensus were staff training, alertness, empathetic attitude of staff towards animals, and facial expressions of pain with 62% of agreement.

### Everyday welfare assessment

**Figure 2:**
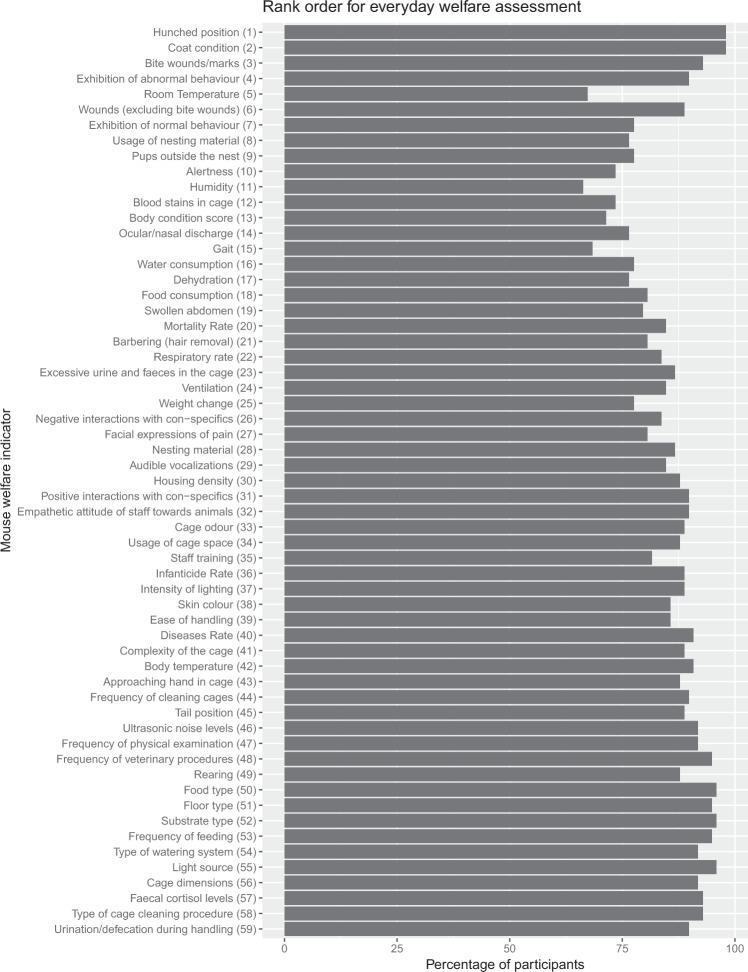
Mean rank order for indicators used in an every-day mouse welfare assessment (final rank position in bracket in front of each indicator). The rank position of the indicators was calculated using the mean of the final position.

Participants were asked to agree or disagree with the rank order of the most important indicators for an everyday welfare assessment by technical staff (Fig. 2). A consensus was reached with 85.7% of agreement between the participants. There were few indicators with consensus level over 95%. These included hunched position, coat condition, food type, substrate type and light source. Humidity, room temperature and gait were the indicators with the lowest consensus level with 66%, 67% and 68% respectively.

### Top ten indicators to be used in a half day audit assessment and in an everyday welfare assessment

**Figure 3:**
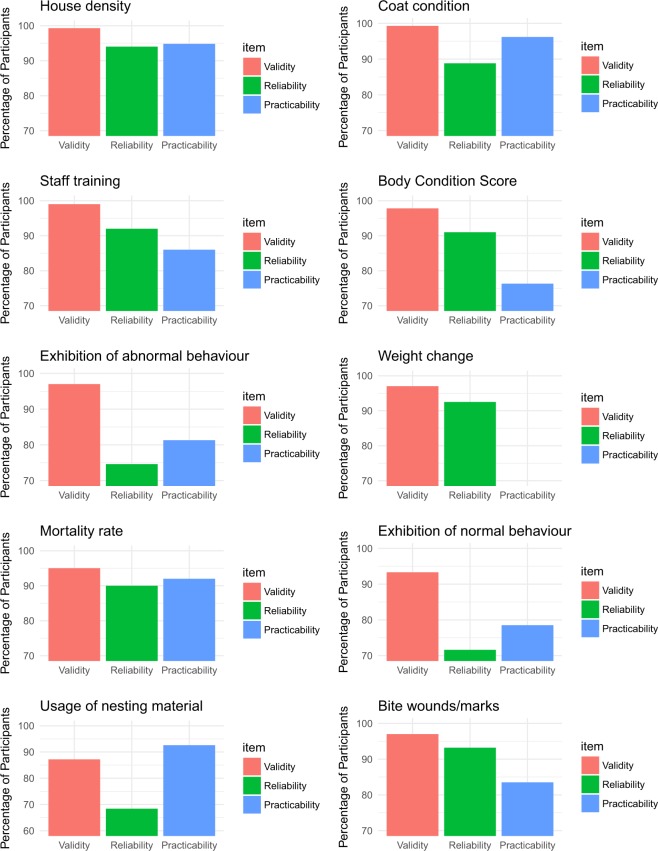
Summary of top ten indicators selected by participants to be used in an audit assessment. The x-axis represents the three items that were assessed by participants, validity (red), reliability (green) and practicability (blue). The y-axis represents the percentage of participants who scored each item as valid and very valid in the Delphi consultation.

**Figure 4:**
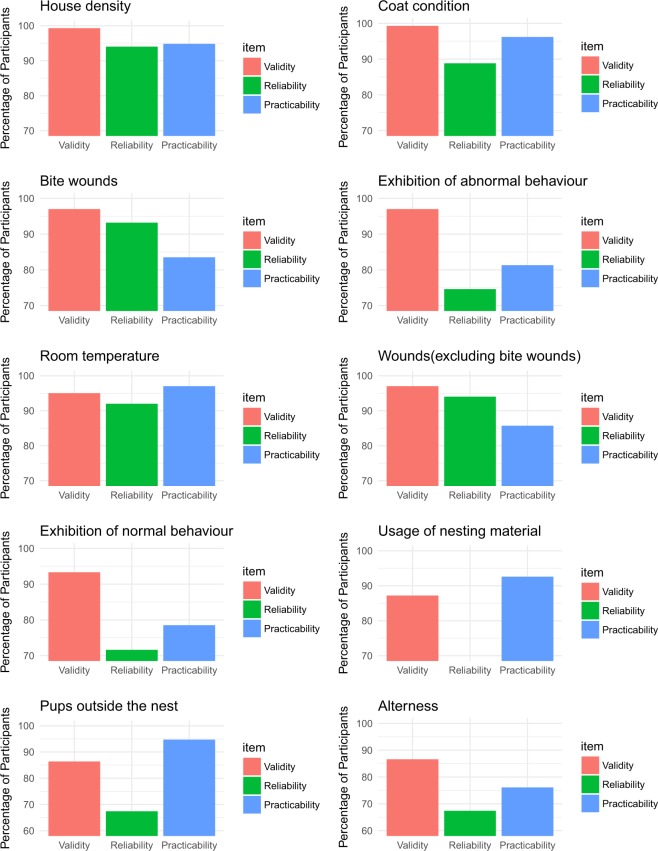
Summary of the top ten indicators selected by participants to be used in an everyday welfare assessment. The x-axis represents the three items that were assessed by participants, validity (red), reliability (green) and practicability (blue). The y-axis represents the percentage of participants who scored each item as valid or very valid in Delphi consultation.

**Figure 5:**
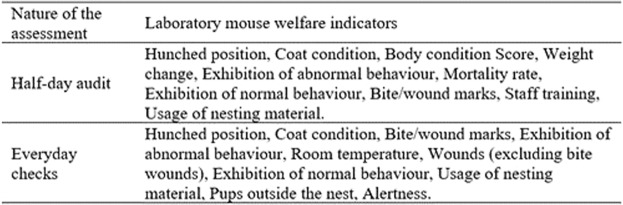
Final top ten indicators list for the laboratory mouse welfare assessment in a half-day and everyday scenarios obtained after the Delphi consultation.

The final top ten list of indicators with the information regarding the percentage of validity, practicability and reliability from both half day animal welfare audit and everyday welfare assessment are provided in Figs 3 and 4 respectively. The percentage validity, reliability and practicability of the indicators included in both half-day and everyday welfare assessment vary between the indicators. All of the indicators show validity over 80% in both, half-day and every day, however, reliability and practicability were different. In the half day welfare assessment, most of the indicators had over 80% reliability except exhibition of normal (74.6%) and abnormal behaviour (71.6%) and usage of nesting material (68.4%). Practicability was under 80% for Body Condition Score (76.3%), weight change (59.7%) and exhibition of abnormal behaviour (78.5%). In the everyday welfare scenario, reliability was under 80% for exhibition of abnormal behaviour (71.6%), usage of nesting material (68.4%), pups outside the nest (67.4%) and alertness (67.4%). Practicability was under 80% for exhibition of abnormal behaviour (78.5%) and Alertness (76.1%). The selection of some of these final top ten indicators for the half day audit or everyday welfare assessment (Fig. 5) were associated with participant’s experience in working with laboratory animals. There is a positive association between the selection of body condition score as top ten indicator and the length of time that participants have been working with laboratory animals (*x*2 = 14.4; *p* = 0.02). This indicator was chosen more by people with over 6 years of experience (48%). Similarly, hunched position (x2 = 12.2; *p* = 0.01) and mortality rate (x2 = 10.4; *p* = 0.03) seems to be positively associated with the length of time that participants have been working with laboratory mice. These indicators were chosen more frequently (50%) by  participates with over 6 years of experience working with laboratory animals.

## Discussion

The aim of this study was to determine, through expert opinion, which laboratory mouse welfare indicators would be valid to use in a half-day welfare audit of a laboratory mouse facility and in an everyday welfare assessment carried out by technical staff.

**Figure 6:**
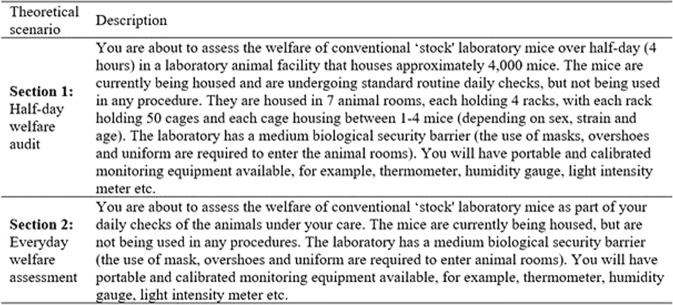
Theoretical scenarios used in the Delphi consultation process for the assessment of validity, reliability and practicability of the 59 laboratory mouse welfare indicators. The two scenarios involved a half-day and everyday welfare assessment.

Delphi methodology has been shown to be a valuable tool for aggregating information from laboratory mouse welfare experts across the world, allowing experts to exchange opinions and come to a consensus. The Delphi consultation process focused on the rank order of 59 indicators in each specific context (see material and methods Fig. 6). Consensus was reached with an agreement of 70% for the top ten indicators for a half-day welfare audit assessment (see Fig. 5). The highest ranked three indicators with the highest agreement (over 84%) did not change position from round one, supporting participant’s opinion about the high validity of these three indicators. Most of the indicators are animal-based (8 out of 10) demonstrating the high credibility (or the high level of confidence) that this type of indicators has between the participants. It is interesting to note that the top four indicators are physiological, followed by indicators relating to behaviour (normal and abnormal), social interaction and the environment. These results further support the idea of the importance of physiological indicators in welfare assessment^34^. These physiological responses which constantly adapt to maintain animal’s welfare can be measured in a non-invasive manner, which might provide a high level of validity^35^. Behavioural indicators are also important as they are easy to measure, and they show an animal’s adaptations to present environmental conditions^7,36,37^.

Conversely, mortality rate and staff training are the only two resource-based indicators included in this list. Staff training can have a significant impact on laboratory mouse welfare as inadequate training can lead to improper care of animals, e.g. handling which can cause fear affecting animal’s performance and welfare^38,39^. Despite the small number of resource-based indicators selected, their inclusion in welfare assessment protocols is important as they include procedures, treatments and management which can have a high welfare impact, especially in laboratory animals (e.g. room temperature preferences, environmental enrichment in the cages)^11^. This is contrary to other authors suggestions  that the assessment of welfare should focus on only animal-based indicators^40^.

The indicators with the lowest percentage of agreement (62%): staff training, alertness, empathetic attitude of staff towards animals and facial expressions of pain were still highly ranked (9, 11, 12 and 16 out of 59 respectively). These indicators are considered to be subjective as it is the observer who gives a score based on observations. This subjectivity might explain their high rank but low general agreement. The fact that participants considered these indicators as important, despite not yet being fully validated, as some of them are relatively new (e.g. facial expression of pain – method initially published by Langford, *et al*.^41^ in 2010) might also have a role in their rank position. In addition many of the studies of the MGS conducted to date, have used it for retrospective scoring from either video or still images. The limited number of studies that have used the MGS for ‘live’ scoring have shown conflicting results, with some finding similar scores to retrospective scoring^42^ and some finding retrospective scores to be higher than those of live scores^43^. This potential inconsistency when scoring live compared to retrospectively may have lead the participants to be concerned about the practicability and reliability of this method at the cage-side and so influenced the ranked position it was given by the participants^41,44,45^.

The top ten indicators selected for an every-day welfare assessment can be seen in Fig. 5. The consensus reached for all the indicators was higher (87.5%) compared to the half-day audit assessment rank order. One possible explanation for  the difference in  the agreement for both scenarios could be due the nature of the assessment, one for auditing purpose (half-day welfare assessment) and the other for daily check-ups (every day welfare assessment).

An important finding of this study is the differences between the final lists for each scenario. Even though they come from the same initial list of 59 indicators they differ by 4 indicators. The top ten half-day audit welfare assessment indicators include: body condition score, weight change, mortality rate and staff training which are not present in the every-day welfare assessment top ten. These differences could be explained in part by the nature of the assessment (i.e. the scenario) proposed in the questionnaire (see material and methods: Fig. 6). Even if the scenario was the same for these two assessments, there are differences in the assessment duration and the individual who is performing the assessment. An everyday welfare assessment, for example, is usually performed by technical staff, who have knowledge about the facility and the animals being assessed. In order to comply with the time limit for a half day audit (4 hours) the indicators used need to be accurate, practical and rapid to score therefore indicators such as body condition score, mortality rate and staff training were deemed relevant by the experts. Body condition score, for example, provides information about mouse health status in a more practical manner than assessing body weight, where a scale and comparison of previous weight is needed^46^. Mortality rate is a resource-based indicator used as a retrospective assessment of welfare as provided information about the number of animals found  dead (i.e. Diseases, environmental problems)^47^. However, this indicator is not considered as a welfare measure because it is performed at facility level thus it is not an indicator of individual welfare^48^ that would be used to assess the welfare on daily basis. In contrast to farmed animals where mortality rate is considered a useful indicator as the level of productivity is directly affected^49^, in laboratory mice mortality rate is not valued in the same manner as it harder to measure at individual level and there are no practical implications for improving welfare state of that individual. Staff training is also an important indicator for a half-day welfare audit where a  longitudinal approach to welfare is considered. Although there is limited research about the real impact of the staff training on the welfare of laboratory mice, recommendations about laboratory animal welfare consider the ability to handle, train and observe mice in the laboratory can be very important to reduce negative impacts on welfare as experienced and trained staff can identify problems promptly^50^.

Alternatively, room temperature, wounds (excluding bite wounds), pups outside the nest and alertness are included in the every-day welfare assessment top ten list but not in the half-day audit assessment. As discussed, room temperature, wounds and alertness are important for the assessment of laboratory mouse welfare. The usage of these indicators in every-day welfare assessment is likely relevant as the assessment is made daily using records (room temperature) or observing the animals by technical staff in the daily welfare check (pups outside the nest, wounds and alertness). Due to the fact that the staff who perform this assessment are in contact with the animals every day, they are likely to be effective at noticing subtle changes such as these more quickly. The staff are already familiar with the species, the strain, the individuals, and in many cases the protocol procedures, therefore they are more experienced in assessing these indicators.

It is important to emphasise that even though this study uses a rank order to define the level of face validity, considering expert opinion, rank order is not relevant for the indicators in terms of defining their individual level of importance over other indicators (i.e. meaning that 10 is not less important than 9). The importance  of this study is in identifying the final list of indicators, considering the type of assessment scenario, and not the assessment of each individual indicator. As it has been stated before, it is an aggregation of different indicators into a protocol which determines the value of the final welfare assessment and not a single indicator alone^48,51–54^. It is important also to highlight the variation in  validity, practicability and reliability between the final top ten indicators for both assessments. The percentage of validity for all the indicators was over 80% which supports their inclusion in the final list as the most valid indicators taking into account expert opinion. However, the reliability and practicability of the indicators was variable between the different measures. Reliability was under 80% for exhibition of normal and abnormal behaviour and usage of nesting material for the half-day welfare assessment. In the everyday welfare assessment, reliability was low for exhibition of abnormal behaviour, usage of nesting material, pups outside the nest and alertness. Although the values of reliability were over 70%, which was the threshold for accepting the agreement between the experts, their lower values of reliability show that these indicators are considered valid and relevant, but their reliability needs to be taken into account and potentially investigated further. The assessment of laboratory mouse behaviour (including normal, abnormal and usage of nesting material) can be a valuable tool for the assessment of welfare and it has been used in other protocols before^40^, but requires a lot of practice and knowledge for the assessor to use effectively and so the results may not be viewed as entirely reliable, particularly as laboratory mouse behaviour can be affected by the presence of an observer^38^. Practicability was considered as low for exhibition of abnormal behaviour, body condition score and weight change in the half-day welfare assessment and for exhibition of abnormal behaviour and alertness in the everyday welfare assessment. This low percentage of practicability (under 80%) for these indicators in both assessments, shows that even if these indicators are considered valid and so important, they are not viewed as very practical. This may be related to the need for baseline recordings to make these indicators truly effective which could have affected how the practicability of these indicators was viewed by the assessors.

Some caution should be taken in interpreting the results from this study. The scenarios used involved a specific description of facilities which can affect the indicators selected as well as the purpose of the selection. Due to the nature of the suggested scenarios and the specific information about the facilities (number of animals, racks, room, etc.), a specific list of indicators have been selected which may not be applicable in different circumstances. It is important to highlight that the indicators selected for this study are those that relate to the influence on welfare of housing and husbandry rather than indicators related to the experiments conducted in animals, which were not included. However, these specific procedure indicators are important in laboratory mouse welfare as procedures (e.g. surgeries, treatments, and behavioural tests) have a direct impact on welfare, affecting physical and psychological health which need to be measured using specific indicators (e.g. Body Condition Score for assessing mouse condition in tumours studies)^55–58^.

This study has several practical implications. It could be used as a preliminary source of face validation to select indicators for a mouse welfare assessment considering the purpose of the assessment, i.e. a welfare audit or daily welfare check. Furthermore, this study illustrates that more research regarding validation, reliability and practicability of welfare indicators to assess laboratory mouse welfare is needed. It also can be concluded that when assessing stock mice, or those not yet actively enrolled on research protocols, the indicators of welfare in Fig. 5 are deemed the most valid to use, based on expert opinion, considering the nature of the assessment (audit welfare assessment or everyday welfare checks). An example score sheet for the audit welfare assessment and everyday welfare check can be found in the supplementary documents. The indicators in each of the example score sheets, which include both resource-based and animal-based indicators, can be used as a preliminary tool for designing a mouse welfare protocol for stock animals in a laboratory facility. Additionally, these indicators could be used as a preliminary list when assessing the welfare of mice enrolled on scientific studies with the addition of key information from study plans and project licenses. These additional experiment specific indicators can be added to an assessment protocol aligned with a preliminary definition of good welfare for the animals being assessed.

## Methods

### Ethical statement

This study were conducted at Newcastle University following the registration for unlicensed work (AWERB Project ID: 449), and in accordance with the EU Directive (2010/63/EU), and  ASPA (1986). An informed consent was obtained from all participants.

**Figure 7:**
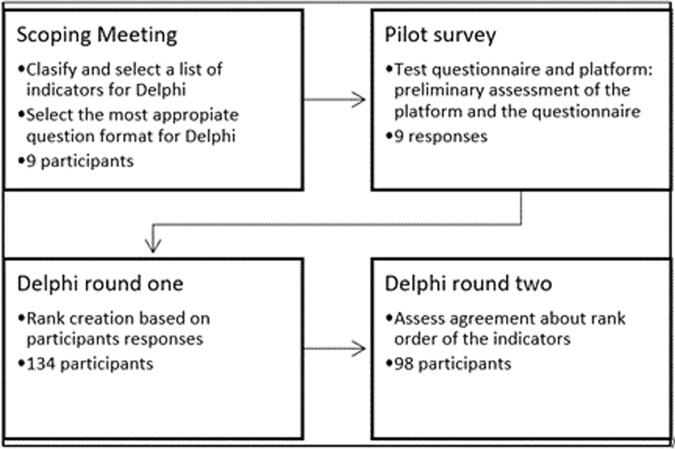
Delphi consultation process which comprises four sequential phases carried out for this study. The phase one was the scoping meeting, followed by phase two (pilot survey), phase three (Delphi round one), and finally phase four (Delphi round two).

A Delphi consultation process was conducted to determine, through expert opinion, the most valid, reliable and practical indicators to be use in a half day and every day assessment of laboratory mice welfare. It comprised of four distinct sequential phases; [1] a scoping meeting, [2] a pilot survey, [3] Round one of Delphi consultation, and [4] Round two of Delphi consultation (Fig. 7).

The scoping meeting was divided into two sessions. In session one participants were asked to generate as many indicators of mouse welfare as possible, considering their validity, reliability and practicability for the assessment of mouse welfare in a specific context. In session two, the groups were asked to rank the quality of a list of potential questions that could be used in the first round of Delphi consultation. The pilot survey of the first round of the Delphi consultation was launched using the on-line system Qualtrics platform (http://www.qualtrics.com) and was live for 2 weeks (December 2015) with 9 participants completing it. Participants were asked to complete the survey and assess the type of questions, their clarity and to indicate the amount of time needed to complete the questionnaire. This survey provided feedback on the type of questions, the length and the information contained within the questionnaire, and was used to refine the questions and survey format for the first round of the Delphi consultation.

### First-round questionnaire

Participants were recruited using a diverse set of methods, including personal contacts, professional organisations relating to laboratory animal welfare, veterinarians working in laboratory animal facilities, literature search of academics and researchers who have published on mouse welfare in the last 15 years. A total of 206 people agreed to take part in this first round. The questionnaire was then sent via an individual link to each participant using Qualtrics platform (http://www.qualtrics.com). Consenting participants were informed about the aim, methods and duration of the study. Data collected was only used for this specific research project. Ethical approval was granted from Newcastle University (Project ID 449).

The questionnaire was separated into 3 sections. In the first two sections, the participants were given two different theoretical scenarios as a guide to complete the questions relating to the validity, practicability and reliability of potential welfare indicators (Fig. 6). The indicators were divided into two separate groups, animal-based and resourced-based indicators. This was done to facilitate the assessment process in the Delphi consultation round one questionnaire. Validity was defined as ‘an indicator that provides useful information about the animal’s welfare’; practicability was defined as ‘an indicator that can be measured in a reasonable amount of time, incurring a reasonable cost and is feasible within the constraints of a laboratory animal facility’; and reliability was defined as ‘an indicator that produces consistent information when used by different people assessing the same animal and the same person assessing the same animal in the same state on more than one occasion’. This questionnaire was ‘live’ for two weeks (February 2016). The rank order was created from the indicators assessed as ‘valid’ and ‘very valid’ by the participants. Those indicators were then organised into a rank according to how frequently they were selected by participants from 1 to 59.

### Second-round questionnaire

The round two questionnaire was sent out to the participants who completed the round one questionnaire using the Qualtrics platform with a personal link via email. This questionnaire was again ‘live’ for two weeks (March 2016). The second-round questionnaire was separated into two sections in which the participants assessed the rank order of the indicators for both scenarios (half-day and everyday assessment). Participants were instructed to agree or disagree with the rank order of all 59 indicators (included both animal- and resource- based measures) taking into account their validity, reliability and practicability for the assessment of laboratory mouse welfare in each scenario. If they disagreed, they were then asked to reorder the indicators into the rank position they considered more appropriate and state the reason for the change (i.e. based on validity, practicability and/or reliability). The above process was repeated for scenario for section 2 (everyday welfare assessment). We chose to include both animal- and resource-based measures together to determine the ones the participants felt were the most important for assessing welfare within the constraints of the time available for carrying out such assessments.

### Data analysis

Information about participant’s selection in validity, practicability and reliability of the indicators was analysed using descriptive statistics. The Delphi consultation methodology is a qualitative method use for gathering information about people’s opinion. Most of the research performed using this methodology used descriptive statistics (frequencies, means, median) for analysing the data^12–14,18,59^. There is also research comparing different Delphi techniques and providing advice about the analysis which recommend the usage of frequencies, mean, median for analysing data and provide final results^15,17,27,28,60–62^. Participant’s selection in terms of the validity of the indicators and rank order were compared across participant’s job role and years of experience working with laboratory animals using a Chi-square test. These two factors (job role and years of experience) were chosen out of the 8 factors from the demographic information of the participants because they are representative of the level of experience with laboratory mice which is considered as an important factor for defining participant’s expertise in the area.

## Supplementary information

Supplementary information

## Data Availability

The datasets generated during and/or analysed during the current study are available from the corresponding author on reasonable request.
