# Supplementary material for: Validation of mouse welfare indicators: a Delphi consultation survey
Source: Sci Rep. 2019 Jul 15;9:10249. doi: 10.1038/s41598-019-45810-y (PMC6629659; doi:10.1038/s41598-019-45810-y)
Supplement: Supplementary file 1 — Supplementary information [file 41598_2019_45810_MOESM1_ESM.pdf]

Validation of mouse welfare indicators: a Delphi consultation survey

Ivone Campos-Luna[1,*], Amy Miller[1], Andrew Beard[1], and Matthew Leach[1]

[1]School of Natural and Environmental Sciences, Agriculture Building, Newcastle University, Newcastle upon Tyne, NE1 7RU, UK
[*]i.campos-luna@newcastle.ac.uk

Supplementary information

Example score sheets for the audit welfare assessment and everyday welfare assessment are shown below. Qualitative Behavioural Assessment (QBA) is a qualitative indicator that was included in the final score sheet list but was not part of the Delphi consultation. It was additionally included because the results of the intra- and inter-observer reliability showed promising results in the usage of QBA for the assessment of laboratory mouse welfare. QBA takes into account the observer's description of what they see and interpretation of the animal's overall demeanour integrating the animal's subjective experience and its behaviour by focusing on the animal as a dynamic and communicative whole agent[1] . This animal expressivity considers categories such as curious, timid, calm, excited as the description of behavioural styles or expressions, which provides more information about the animal 1. Each term was defined and assessed using a Visual Analogue Scale. The validation of QBA, assessing its inter- and intra-observer reliability for use in laboratory mice has previously been demonstrated[2].

Audit welfare assessment protocol-score sheets

FACILITY ASSESSMENT
DATE:
FACILITY:
START TIMES: FINISH TIME:

Welfare indicator	Scale	SCORE
Temperature	Current room temperature	
Humidity	Current room humidity	
Ventilation	Current room/cage ventilation	
Handling method	Proportion of animal handled by tail	
	Proportion of animals handled by cupping	
	Proportion of animals handled by tunnel	
Frequency of cleaning cages	Frequency of cleaning cages	
	Number of air changes per hour in the cages	
Type of cleaning procedure	Mice are transferred to a clean cage with clean sawdust and the nesting material from the dirty cage	
	Mice are transferred to a clean cage with clean sawdust, dirty sawdust for the dirty cage and clean nesting material	
	Mice are transferred to a clean cage with clean sawdust and clean nesting material.	
Staff training	Mainly all the staff have Professional Development Scheme (PDS) related to the care of laboratory animals (more than 51%)	
	There is a proportion (30 % to 50%) of the staff that have PDS	
	Staff do not have PDS (less than 29%)	

CAGE ASSESSMENT

START TIME: FINISH TIME:

CAGE ID:

Welfare indicator	Scale	score
Complexity of the cage	Additional elements or structures present	
	Only nesting material as additional element	
	No additional material or elements	
Alertness	Mouse alert when cage lid is open	
	Mouse might respond to the observer but not immediately.	
	Mouse does not show any interest in the environment or the observer.	
Use of nesting material	Complete or incomplete dome	
	A cup-shaped or flat nest	
	Untouched or scattered nesting material throughout the cage	
Qualitative Behavioural assessment (QBA)[1]	See next page	
Blood stains in the cage	Blood stains absent in the home cage	
	Blood stains present in the home cage	

QUALITATIVE BEHAVIOURAL ASSESSMENT

CAGE ID:

DATE:

	Min.	Max.
Inquisitive	Min.	Max.
In pain	Min.	Max.
Positively Engaged	Min.	Max.
Lethargic	Min.	Max.
Energetic	Min.	Max.
Depressed	Min.	Max.
Determined	Min.	Max.
Anxious	Min.	Max.
Confident	Min.	Max.
Agitated	Min.	Max.
Calm	Min.	Max.
Fearful	Min.	Max.

Content
Min. Max.
Tense
Min. Max.
Comfortable
Min. Max.
Uncertain
Min. Max.
Playful
Min. Max.
Bored
Min. Max.
Sociable
Min. Max.
Frustrated

INDIVIDUAL ASSESSMENT
START TIME: FINISH TIME:
CAGE ID
MOUSE ID

Indicator	scale	Score
Hunched posture	No hunched posture present	
	Walks slowly in a hunched posture	
	Hunched posture with no movement	
Coat condition	Shiny, clean and well-groomed coat	
	Coat clean but ungroomed	
	Ruffled and untidy coat, it may be greasy and stick together	
Gait	Body weight supported on all limbs	
	Mouse might limp while walking	
	Mouse has difficulty moving forward or is reluctant to walk	
Ocular/nasal discharge	No ocular/nasal discharge	
	Water-like or mucus-like ocular/nasal discharge	
Wounds/marks (including bite wounds)	No wounds	
	Superficial Wounds	
	Extensive and deep wounds	
Body Condition Score	The mouse is well-conditioned	
	The mouse in either under-conditioned or overweight	
	The mouse is either emaciated or obese.	
Barbering (hair removal)	No barbering	
	Barbering on different parts of the body	
Swollen abdomen	Abdomen is not swollen	
	Abdomen moderately swollen	
	Abdomen is very swollen	

Everyday welfare assessment protocol-score sheets

FACILITY/CAGE ASSESSMENT:
FACILITY:
CAGE ID:

Indicator	Scale	Date				
Temperature	Current room temperature					
Humidity	Current room humidity					
Ventilation	Current room/cage ventilation					
Complexity of the cage	Additional elements or structures present					
	Only nesting material as additional element					
	No additional material or elements					
Alertness	Mouse alert when cage lid is open					
	It might respond to the observer but not immediately.					
	Mouse does not show any interest in the environment or the observer.					
Use of nesting material	Complete or incomplete dome					
	A cup-shaped or flat nest					
	Untouched or scattered nesting material throughout the cage					
Qualitative Behavioural assessment (QBA)	See next page					
Blood stains in the cage	Blood stains absent in the home cage					
	Blood stains present in the home cage					

QUALITATIVE BEHAVIOURAL ASSESSMENT
CAGE ID:
DATE:

	Min.	Max.
Inquisitive		
	Min.	Max.
In pain		
	Min.	Max.
Positively Engaged		
	Min.	Max.
Lethargic		
	Min.	Max.
Energetic		
	Min.	Max.
Depressed		
	Min.	Max.
Determined		
	Min.	Max.
Anxious		
	Min.	Max.
Confident		
	Min.	Max.
Agitated		
	Min.	Max.
Calm		
	Min.	Max.
Fearful		
	Min.	Max.
Content		
	Min.	Max.
Tense		
	Min.	Max.
Comfortable		
	Min.	Max.
Uncertain		
	Min.	Max.
Playful		
	Min.	Max.
Bored		
	Min.	Max.
Sociable		
	Min.	Max.
Frustrated		

INDIVIDUAL ASSESSMENT
CAGE ID
MOUSE ID

Indicator	Scale	Date				
Hunched position	No hunched posture present					
	Walks slowly in a hunched posture					
	Hunched posture with no movement					
Coat condition	Shiny, clean and well-groomed coat					
	Coat clean but ungroomed					
	Ruffled and untidy coat, it can be greasy and stick together					
Gait	Body weight supported on all limbs					
	Mouse might limp while walking					
	Mouse has difficulty moving forward or is reluctant to walk					
Ocular/nasal discharge	No ocular/nasal discharge					
	Water-like or mucus-like ocular/nasal discharge					
Wounds/marks (including bite wounds)	No wounds					
	Superficial wounds					
	Extensive and deep wounds					
Body Condition Score	The mouse is well-conditioned					
	The mouse in either under-conditioned or overweight					
	The mouse is either emaciated or obese.					
Barbering (hair removal)	No barbering					
	Barbering on different parts of the body					
Swollen abdomen	Abdomen is not swollen					
	Abdomen moderately swollen					
	Abdomen is very swollen					

List of the fixed behavioural expressions for assessing the emotional states of laboratory mice using Qualitative Behavioural Assessment

Term	Definition
Inquisitive	The mouse appears curious and interested in others and in exploring the environment. Willing to investigate.
Positively engaged	The mouse is carrying out activities in a focused, directed and constructive manner. The mouse appears not to be distracted by others or the environment.
Energetic	The mouse is carrying out an activity with a lot of energy and vigour, in a lively and excited manner.
Determined	The mouse is showing an active and rapid reaction to something or someone. It appears to be focused on accomplishing a specific goal or task.
Confident	The mouse is displaying assertiveness, behaving assertively with other animals and its environment in a self-assured manner.
Calm	The mouse appears peaceful and without worry. The mouse behaves in a relaxed and untroubled manner.
Content	The mouse appears happy and satisfied. Expressing happiness, with all its physiological, environmental and psychological needs met.
Comfortable	The mouse appears physically satisfied with the cage environment and looks relaxed and free from discomfort.
Playful	The mouse is engaging in lively movements purely to frolic or for fun, expressing pleasure, happiness and amusement.
In Pain	The mouse is suffering from physical discomfort leading the mouse to be reluctant to move, or to move with abnormal gait, or showing a tense, hunched or uncomfortable posture. The mouse looks like it is hurting or suffering and is in discomfort.
Lethargic	The mouse appears fatigued and sluggish. It has a lack of vigour and energy, showing low amounts of movement and any movement is slow and ponderous.
Depressed	The mouse appears unhappy and without hope. It is apathetic, despondent and unresponsive showing little or no response or reaction to anyone or its environment. It appears isolated.
Anxious	The mouse is uneasy, cautious and nervous
Agitated	The mouse appears to be irritable and highly reactive. An excess of physical and cognitive activity is present because of anxiety.
Sociable	The mouse actively interacts with others. It is willing to interact with others showing affiliative actions (e.g. grooming, resting in groups, sniffing)
Fearful	The mouse appears afraid or scared. It seems to be avoiding contact with others and the environment looks to be hiding, looking for a way out or trying to escape.
Tense	The mouse looks worried and emotionally tense. Its posture might evidence physical tension.
Uncertain	The mouse appears to be insecure; its physical movement is cautious. The slowly showing alertness and insecurity. Avoidance reactions are showing with all stimuli

Bored	The mouse appears uninterested in its environment and/or cage mates. The way it moves around and orients itself appears to be unfocused and aimless, without much energy, never staying long with a particular activity or aspect of the environment.
Frustrated	The mouse appears unfulfilled with its environment and/or cage mates. It looks stressed and uneasy showing repetitive and fast movements.

References

1 Wemelsfelder, F. How animals communicate quality of life: the qualitative assessment of behaviour. *Anim Welfare* **16**, 25-31 (2007).

2 Campos-Luna, I. *The development of a quantitative and qualitative protocol for assessing the welfare of laboratory mice* PhD thesis, Newcastle University, (2019).
